# Effects of rumen‐protected methionine on milk production in early lactation dairy cattle fed with a diet containing 14.5% crude protein

**DOI:** 10.1111/asj.13123

**Published:** 2018-11-05

**Authors:** Tetsuo Tamura, Kazunori Inoue, Hideto Nishiki, Masafumi Sakata, Makoto Seki, Teruaki Koga, Yoshihiro Ookubo, Kazuhiro Akutsu, Say Sato, Kouichi Saitou, Hikari Shinohara, Terumi Kuraisi, Hiroshi Kajikawa, Mitsunori Kurihara

**Affiliations:** ^1^ Tokyo Metropolitan Agriculture and Forestry Research Center Ome Japan; ^2^ Niigata Agricultural Research Institute Livestock Research Center Sanjo Japan; ^3^ Nagano Animal Industry Experiment Station Shiojiri Japan; ^4^ Tochigi Prefectural Livestock and Dairy Experimental Center Nasushiobara Japan; ^5^ Aichi Agricultural Research Center Nagakute Japan; ^6^ Chiba Prefectural Livestock Research Center Yachimata Japan; ^7^ Gunma Prefectural Livestock Experiment Station Maebashi Japan; ^8^ Yamanashi Prefectural Stock Raising Farming Technology Center Nagasaka Branch Hokuto Japan; ^9^ National Institute of Livestock and Grassland Science NARO Tsukuba Japan

**Keywords:** crude protein, dairy cow, metabolizable methionine, rumen‐protected methionine

## Abstract

We evaluated the influence on milk production of feeding early lactation cows a diet that included 14.5% crude protein (CP) and that did not meet methionine (Met) requirements or that met them by supplying rumen‐protected Met (RPMet). Thirty‐nine multiparous Holstein cows were allocated into two groups. For 15 weeks after calving, each group was fed one of the two total mixed rations, Control (*n *=* *20) or Treatment (*n *=* *19). The Treatment group received added RPMet at 0.034% (8 g/day) of the Control diet on dry matter basis. The adequacies of Met for the Control and Treatment groups were 96% and 106%, respectively, and for other amino acids, >110%. The CP level (14.5%) was 1 percentage point lower than that recommended by the Japanese Feeding Standard (2006). No between‐group differences were found in milk yield (40 kg/day), milk composition, plasma profile, rumen fermentation, nitrogen balance, or cow health. Met intake and the amount of rumen‐undegradable feed Met were higher in the Treatment group (*p *<* *0.05). Microbial Met and total metabolizable Met did not differ between groups. Supplying RPMet in a 14.5% CP diet during early lactation did not dramatically affect milk production, because the amount of total metabolizable Met was unchanged.

## 1 INTRODUCTION

In dairy cattle, lysine (Lys) and methionine (Met) have been identified most frequently as the first‐limiting essential amino acids in metabolizable proteins (NRC, 2001; Xu et al., 1998). Met is more limiting than Lys, and cows have responded favorably to supplementation with rumen‐protected Met (RPMet) (Socha et al., 2005). It is therefore well known that milk production can be improved by feeding RPMet. One meta‐analysis has revealed that RPMet supplementation enhances the percentage and yield of true milk protein (Patton, 2010). RPMet supplementation increases milk yield through increased voluntary dry matter intake (DMI) and enhanced liver function (Batistel et al., 2017). RPMet improves postpartum lactation performance and enhances immune function in transition dairy cows (Sun et al., 2016).

The dietary level of crude protein (CP) is one of the most important factors in milk production. The amount of nitrogen (N) in dietary CP that is excreted in manure is about two to three times the amount excreted in milk (Broderick, 2003). Overfeeding of CP results in an energy cost to the animal; this cost is associated with the conversion of excess protein to urea (Dinn, Shelford, & Fisher, 1998). Despite a decrease in dietary CP content, milk production and composition were unchanged in one study (Bahrami‐Yekdangi, Ghorbani, Khorvash, Khan, & Ghaffari, 2016). Reducing the intake of CP and rumen‐degradable protein can decrease blood plasma urea levels (Gordon & McMurray, 1979; Westwood, Lean, Garvin, & Wynn, 2000) and is favorably associated with conception rate (Ferguson, Galligan, Blanchard, & Reeves, 1993). According to the Japanese Feeding Standard (JFS), 15.5% dietary CP is an optimal level during early lactation (NARO, 2006). Nevertheless, we have found previously that diets with 17.5%–14.5% CP and 8.0%–5.0% rumen‐undegradable protein (RUP) do not affect milk production (unpublished). The amounts of N in milk and feces were not altered, whereas the amount of urinary N was increased, when cows were fed diets containing more than 14.5% CP and 5.0% RUP. We concluded that 14.5% CP, 5.0% RUP, and 9.5% rumen‐degradable protein gave an optimum dietary N level. Similarly, Bahrami‐Yekdangi et al. (2016) reported that approximately 9.5% rumen‐degradable protein in diets provided sufficient protein to optimize milk production. However, there is growing concern about the need to validate the Met balance to maintain milk production on a 14.5% CP diet: reducing dietary CP is likely to require carefully tailoring of the dietary amino acid content—particularly the content of Met, as the first‐limiting essential amino acid.

Many studies of RPMet have been performed on diets (e.g., CP 17%, Batistel et al., 2017) that include CP at levels higher than that recommended by the JFS (NARO, 2006). Furthermore, some studies have reported that feeding RPMet to cows in early lactation does not improve production (Misciatteilli et al., 2003; Ohgi et al., 2002).

However, to our knowledge, no experiments have yet investigated the Met balance when diets containing 14.5% CP or lower are fed. Our object here was to quantify the influence on milk production when cows in early lactation were fed a diet that included 14.5% CP—a level lower than that recommended by the JFS—and that met, or did not meet, the Met requirement by supplying RPMet. N. balance and cow health were also investigated.

## 2 MATERIALS AND METHODS

### 2.1 Experimental design, animals, and diets

There were two kinds of experiment: (a) production trials and (b) metabolism trials. Animal care and handling were conducted according to the Fundamental Guidelines for Proper Conduct of Animal Experiment and Related Activities in Academic Research Institutions (Ministry of Education, Culture, Sports, Science and Technology of Japan, Notice No. 71 of 2006).

For the production trials, 39 multiparous Holstein cows from the research organizations of eight prefectures (Tokyo, Niigata, Nagano, Tochigi, Aichi, Chiba, Gunma, and Yamanashi) were assigned to one of two total mixed ration (TMR) diets on the basis of parity and expected calving date. TMRs were of two types: Control (*n *=* *20) and Treatment (*n *=* *19) (Table 1). The Treatment diet included RPMet at 0.034% of the Control diet on a dry matter basis. RPMet was provided as Mepron (Evonik Nutrition and Care GmbH, Essen, Germany). The amount of RPMet was designed to be equivalent to the amount of metabolizable Met in our previous experimental diet (unpublished; our previous diet had similar ingredients and nutrient composition to those of the control diet, 14.5% CP, 5.2% RUP, no added RPMet, but added fish meal as a rumen‐undegradable Met source). According to AminoCow (version 3.5.2, Evonik Nutrition and Care GmbH), for the Control and Treatment groups, the respective adequacies were Met 96% and 106%, and valine 127% and 125%. The adequacies of other amino acids were the same in the two diets: arginine 185%, histidine 115%, isoleucine 130%, leucine 114%, lysine 111%, phenylalanine 144%, threonine 146%, and tryptophan 143%. The two diets were designed to be similar in terms of CP, RUP, neutral detergent fiber (NDFom), acid detergent fiber (ADFom) content, and total digestible nutrients (TDN) content. The level of CP was designed to be 14.5%—one percentage point lower than the value recommended by JFS (NARO, 2006). TDN was adjusted to meet the requirements of JFS (NARO, 2006). Each cow was fed the diet 3 weeks before the expected date of parturition (3‐week habituation period) and then entered the production trial after parturition; the feeding continued until the end of the metabolism trial (15–18 weeks after parturition). Cows were housed throughout the experiment and were allowed to exercise daily in an outside lot for about 4 h during the day. The TMR was fed at about 09:00 and 16:00 hr. Cows had free access to the ration. Cows were milked twice daily.

**Table 1:** Ingredient and nutrient compositions of diets

Item	Diet	
	Control	Treatment^a^
Ingredient, % of dry matter		
Timothy hay	14.54	14.53
Alfalfa hay	14.54	14.53
Steam‐rolled maize	21.09	21.08
Barley	11.05	11.05
Cottonseed with lint	8.98	8.98
Beet pulp	10.16	10.16
Soybean meal	3.22	3.22
Soybean hull	5.11	5.11
Wheat bran	4.96	4.95
Corn gluten feed	3.04	3.04
Molasses sugarcane	1.66	1.66
Mepron	‐	0.03
Mineral and Vitamin mix^b^	0.26	0.26
Calcium carbonate	0.61	0.61
Dicalcium phosphate	0.45	0.45
Salt	0.34	0.34
Nutrient composition, % of dry matter		
CP	14.3	14.3
RUP^c^	5.0	5.0
Ether extract	3.6	3.5
NDFom	37.1	37.0
ADFom	23.8	23.1
Crude ash	6.6	6.8
Nonfibrous carbohydrate	38.6	38.6
Total digestible nutrients^c^	75.6	75.6
Amino acids composition, g/kg of dry matter		
Arginine	7.65	7.64
Histidine	3.20	3.20
Isoleucine	4.63	4.63
Leucine	10.05	10.05
Lysine	6.21	6.21
Methionine	1.85	2.11
Phenylalanine	6.38	6.38
Threonine	5.08	5.08
Valine	6.73	6.72

Control + rumen‐protected methionine. Mepron, rumen‐protected methionine.

A mix of trace minerals (0.44% Fe, 0.35% Cu, 0.46% Zn, 0.33% Mn, 0.005% Co, 0.13% Mg, 0.04% I) and vitamins A, D, and E (2816 IU/g vitamin A, 264 IU/g vitamin D3, and 2.2 mg/g DL‐α tocopherol acetate). CP, crude protein; RUP, rumen‐undegradable protein; NDFom, neutral detergent fiber; ADFom, acid detergent fiber; Nonfibrous carbohydrate [%] = 100 − (CP [%] + Ether extract [%] + NDFom [%] + Crude ash [%]).

Designed value.

Metabolism trials were conducted for 3 days consecutively during a 14‐ to 17‐week period after parturition. The cows used in the metabolism trial were the same as those used in the production trial; they were fed the same TMR as the one to which they had been assigned for the production trial. During the 3 days of sampling for the metabolism trial, the cows used were not released for exercise but were housed in individual tie‐stalls.

### 2.2 Sample collection and data recording

In the production trial, TMR intake and milk yield were recorded daily. Milk samples were collected weekly at two consecutive milkings until 15 weeks. Body weight was measured each week until 15 weeks. Blood samples were collected via the jugular vein approximately 4 h after feeding at 1, 3, 5, 9, and 13 weeks. The samples were placed on ice immediately, and the plasma was obtained by centrifugation. Ruminal juice samples taken from the ventral sac at the time of blood sampling were strained through two layers of cheesecloth. Ruminal fluid pH was measured immediately with a pH meter (F‐22; Horiba Ltd., Kyoto, Japan). Blood plasma and ruminal samples were frozen at −20°C until analyzed.

For the metabolism trial, total feces were collected, TMR intake and total fecal weight were recorded, and TMR and fecal samples were collected daily. Milk yields were recorded, and samples were taken at each milking. Urine samples were taken in one of two ways, namely as total urine samples or as spot urine samples. Total urine was collected with a vulva urine cup (Sanshin Industrial Co. Ltd., Yokohama, Japan) into a container containing 700 ml of 20% (v/v) H_2_SO_4_. Total urine weight was recorded and the sample obtained every day. Spot urine samples were collected from spontaneous urination by manual stimulation of the groin every day. Five milliliters of 20% (v/v) H_2_SO_4_ was added per 100 ml of spot urine. Urine samples were frozen at −20°C until analyzed. Samples (other than the wet samples of feces used for analysis of total N) were prepared as dried samples: wet feces and TMR were dried for 48 h at 55°C, ground, and then passed through a 1‐mm screen.

Health disorder data were recorded from after parturition until the end of the metabolism trial.

### 2.3 Sample analysis and calculations

DMI was measured in the same way as reported in a study by Koga et al. (2001). Samples of TMR and feces were analyzed according to standard methods (AOAC, 1990). Samples of control diet, RPMet, and rumen‐incubated residual (described in the next paragraph as the nylon bag technique) were analyzed for amino acids by using high‐performance liquid chromatography (L‐8500A; Hitachi High‐Technologies Corp.). Gross energy was measured with an adiabatic bomb calorimeter (CA‐4PJ; Shimadzu Corp., Kyoto, Japan). Milk samples were analyzed by using Milko‐Scan (Robert Foss Electric LLC, Hillerød, Denmark). Blood plasma samples were assayed with an automated biochemical analyzer (7040; Hitachi High‐Technologies Corp., Tokyo, Japan). Ruminal samples were analyzed for volatile fatty acids (VFAs) by using a gas chromatograph (GC14A; Shimadzu Corp.), and for ammonia‐N concentration by using the indophenol‐blue method (Novozamsky, van Eck, van Schouwenburg, & Walinga, 1974) and a spectrophotometer (7010; Hitachi High‐Technologies Corp.). Total urine excretion from spot urine was estimated from creatinine and body weight by using the method of Tamura, Inoue, Shinohara, and Koga (2007). Urine samples were analyzed for allantoin, and rumen microbial N outflow into the intestine was determined by using the method of Chen (1989).

Amounts of metabolizable Lys and Met were estimated from the amount of rumen‐incubated residual (as measured by using the nylon bag technique; Lykos & Varga, 1995) and the quantity of absorbable microbial protein in the intestine. The specific method of estimation was as follows: Control diet samples were ground to 2 mm, whereas RPMet samples were left as unground granules. Approximately 7.5 g of control sample or RPMet was placed into a nylon bag. Four bags per sample type (control diet and RPMet) were prepared. The bags were incubated in the rumens of two fistulated cows. The cows were multiparous and in midlactation, and were being fed the Control diet. In each cow, one set of control and RPMet bags was incubated from 06:00 to 18:00 hr and the other set from 18:00 to 06:00 hr. Ruminal disappearance of Lys and Met was calculated as the difference between Lys and Met contents of the initial samples and the residues after incubation in the rumen. We assumed that incubated residing amino acids flowed on to the intestine. The amounts of these amino acids (estimated absorbable rumen‐undegradable feed Met or Lys) were calculated by using DMI and the rate of ruminal disappearance. It was assumed that 80% of the amino acid flow is absorbed (NRC, 1989) and 64% of the microbial CP outflow is absorbed as protein (NRC, 2001). In accordance with the Cornell Net Carbohydrate and Protein System (ver. 4.0.31 model; Fox et al., 2000), the amounts of absorbable microbial amino acids were estimated as 8.20% of the absorbable microbial protein in the case of Lys and 2.68% of the absorbable microbial protein in the case of Met.

### 2.4 Statistical analysis

Data in production trials were analyzed by using the MIXED procedure in SAS (SAS Institute, Inc., Cary, NC, USA) according to the following model:

$$Y_{\mathrm{ijklm}}=\mu +D_{i}+W_{j}+P_{k}+L_{l}+D\times W_{\mathrm{ij}}+C{\left(L\right)}_{\mathrm{ml}}+e_{\mathrm{ijklm}},$$

where *Y*_*ijklm*_ = the dependent variable, μ = the overall mean, *D*_*i*_ = fixed effect of diet (*i *= control or treatment), *W*_*j*_ = fixed effect of week, *P*_*k*_ = fixed effect of parity (*k *=* *2nd or ≥3rd parity), *L*_*l*_ = random effect of experimental location (*l *=* *1 to 8), *D* × *W*_*ij*_ = interaction between diet and week, *C*(*L*)_*ml*_ = random effect of *m*th cow within the *l*th experimental location, and *e*_*ijklm*_ represents the residual error. Data obtained in the metabolism trial were also analyzed by using the model above, but without *W*_*j*_, *P*_*k*_, and *D* × *W*_*ij*_. Daily amounts of urinary allantoin and N were compared between the total urine samples and estimations made from the spot urine samples to determine the validity of the spot sample estimates. Incidences of health disorders were analyzed by using Fisher's exact test with two‐tailed *p* values by using the FREQ procedure in SAS.

## 3 RESULTS

Some cows exhibited health problems such as mastitis and displaced abomasum during the production and metabolism trials. Those cows were excluded. At the end of the production trial, data from 20 Control group cows and 18 Treatment group cows were included in the analyses. In the metabolism trial, data sets from 19 Control group cows and 17 Treatment group cows were used.

### 3.1 Production

Body weight, DMI, and production parameters are presented in Table 2. No item differed between the two groups (*p *>* *0.05). Daily intake of RPMet in the Treatment group was approximately 8 g by DMI (23.7 kg). Milk yield was over 40 kg/day with each diet.

**Table 2:** Body weight, dry matter intake, and production parameters

Item	Diet		*p*		
	Control	Treatment^a^	Diet	Week	Diet × Week
Body weight, kg	658 ± 14	654 ± 15	0.842	<0.001	1.000
Dry matter intake, kg/d	24.5 ± 0.5	23.7 ± 0.6	0.251	<0.001	0.537
Milk yield, kg/d	40.4 ± 1.5	40.5 ± 1.6	0.966	<0.001	0.549
4% FCM, kg/d	37.9 ± 1.7	38.2 ± 1.8	0.855	<0.001	0.509
Milk composition					
Fat, %	3.61 ± 0.18	3.74 ± 0.19	0.270	<0.001	0.655
Protein, %	3.18 ± 0.04	3.09 ± 0.05	0.066	<0.001	0.588
Lactose, %	4.61 ± 0.04	4.59 ± 0.04	0.634	<0.001	0.938
Milk urea nitrogen, mg/dl	11.3 ± 1.1	11.8 ± 1.1	0.079	0.012	0.790
Milk component yield					
Fat, kg/d	1.4 ± 0.1	1.5 ± 0.1	0.730	0.006	0.602
Protein, kg/d	1.3 ± 0.0	1.2 ± 0.0	0.501	0.209	0.571
Lactose, kg/d	1.9 ± 0.1	1.9 ± 0.1	0.980	<0.001	0.855
Milk urea nitrogen, g/d	4.6 ± 0.6	4.8 ± 0.6	0.530	0.001	0.942

Values are least‐squares means ± standard error.

a Control + rumen‐protected methionine. 4% FCM, fat‐corrected milk = (0.4 × kg of milk) + (15 × kg of milk fat).

### 3.2 Blood plasma and Ruminal fluid

Blood plasma profiles are shown in Table 3. Total protein content differed between the two diets (*p *<* *0.05). Ruminal fermentation characteristics are shown in Table 4. The caproate content as a percentage of VFAs differed between the two diets (*p *<* *0.05).

**Table 3:** Blood plasma clinical chemistry profiles

Item	Diet		*p*		
	Control	Treatment^a^	Diet	Week	Diet × Week
Glucose, mg/dl	64.7 ± 1.5	62.8 ± 1.6	0.174	<0.001	0.906
Nonesterified fatty acid, μEq/L	276 ± 45	253 ± 47	0.551	<0.001	0.205
Total cholesterol, mg/dl	185 ± 14	188 ± 15	0.761	<0.001	0.955
Aspartate aminotransferase, IU/L	87 ± 4	83 ± 5	0.398	<0.001	0.830
γ‐glutamyl transpeptidase, IU/L	37.8 ± 2.4	31.6 ± 2.5	0.079	<0.001	0.923
Albumin, g/dl	3.6 ± 0.2	3.5 ± 0.2	0.324	<0.001	0.844
Total protein, g/dl	7.5 ± 0.3	7.1 ± 0.3	0.048	<0.001	0.397
Urea nitrogen, mg/dl	9.7 ± 0.4	10.4 ± 0.4	0.111	<0.001	0.164

Values are least‐squares means ± standard error.

a Control + rumen‐protected methionine.

**Table 4:** Ruminal fluid profiles

Item	Diet		*p*		
	Control	Treatment^a^	Diet	Week	Diet × Week
pH	6.70 ± 0.05	6.63 ± 0.05	0.131	<0.001	0.543
Ammonia‐N, mg/dl	4.6 ± 0.4	5.3 ± 0.5	0.065	0.135	0.648
Total VFA, mmol/dl	8.4 ± 0.3	8.6 ± 0.3	0.174	0.820	0.903
Acetate^b^	59.4 ± 0.7	59.6 ± 0.7	0.823	0.154	0.633
Propionate^b^	25.8 ± 0.9	25.7 ± 0.9	0.855	0.920	0.758
Isobutyrate^b^	1.3 ± 0.1	1.3 ± 0.1	0.598	0.674	0.969
Butyrate^b^	10.1 ± 0.3	10.1 ± 0.3	0.971	0.008	0.959
Isovalerate^b^	1.5 ± 0.1	1.6 ± 0.1	0.205	0.533	0.835
Valerate^b^	1.6 ± 0.1	1.5 ± 0.1	0.334	0.004	0.044
Caproate^b^	0.3 ± 0.0	0.2 ± 0.0	0.012	0.019	0.452
Acetate: propionate	2.37 ± 0.10	2.39 ± 0.10	0.780	0.904	0.640

VFA, volatile fatty acids.

Values are least‐squares means ± standard error.

a Control + rumen‐protected methionine.

b % of total VFA.

### 3.3 Digestibility

Digestibility and TDN are shown in Table 5. All digestibility and TDN did not differ significantly between the two diets (*p *>* *0.10).

**Table 5:** Digestibility and total digestible nutrients

Item	Diet		*p*
	Control	Treatment^a^	
Digestibility, %			
Dry matter	66.8 ± 1.0	68.7 ± 1.0	0.101
Organic matter	68.4 ± 1.0	70.1 ± 1.0	0.152
Nitrogen	61.2 ± 1.4	63.1 ± 1.4	0.174
Ether extract	75.1 ± 3.0	77.0 ± 3.0	0.405
NDFom	51.4 ± 2.1	53.1 ± 2.0	0.388
ADFom	46.2 ± 2.6	48.1 ± 2.5	0.378
Nonfibrous carbohydrate	91.3 ± 1.0	92.1 ± 1.0	0.285
Gross energy	66.0 ± 1.0	67.9 ± 1.0	0.122
Total digestible nutrients, %	70.8 ± 1.1	73.1 ± 1.1	0.125

NDFom, neutral detergent fiber; ADFom, acid detergent fiber.

Values are least‐squares means ± standard error.

a Control + rumen‐protected methionine.

### 3.4 Microbial N and metabolizable Lys and Met

The daily amounts of urinary allantoin and N did not differ between the results obtained from the total urine samples and the estimations made from the spot urine samples (data not shown; urinary allantoin, *p *=* *0.392; urinary N, *p *=* *0.189). After rumen incubation, 18.9% of the Met in RPMet disappeared. Urinary allantoin excretion and microbial N flows to the intestine, and flows of Lys and Met in the digestive tract, are presented in Table 6. Excretion of urinary allantoin and microbial N flows to the intestine did not differ between the two diets (*p *>* *0.10). The amount of estimated total metabolizable Lys did not differ (*p *>* *0.10). The intake of Met and the amount of estimated absorbable rumen‐undegradable feed Met in the Treatment group were significantly higher than in the Control group (*p *<* *0.05). Estimated absorbable microbial Met and estimated total metabolizable Met did not differ between the two groups (*p *>* *0.10).

**Table 6:** Allantoin excretion, microbial nitrogen flow to the intestine, and flows of amino acids in the digestive tract

Item	Diet		*p*
	Control	Treatment^a^	
Allantoin, g/d	79.6 ± 4.8	74.1 ± 5.0	0.217
Microbial nitrogen flow to intestine^b^, g/d	462.5 ± 30.3	428.5 ± 31.5	0.224
Lysine, g/d			
Intake	152.1 ± 3.8	151.2 ± 3.8	0.679
Absorbable rumen‐undegradable feed^b^	72.9 ± 1.8	72.5 ± 1.8	0.646
Absorbable microbial^b^	151.7 ± 9.9	140.5 ± 10.3	0.224
Total metabolizable^c^	225.0 ± 11.0	213.1 ± 11.6	0.304
Methionine, g/d			
Intake	45.2 ± 1.2	51.4 ± 1.3	0.041
Absorbable rumen‐undegradable feed^b^	26.1 ± 0.7	30.5 ± 0.7	0.032
Absorbable microbial^b^	49.6 ± 3.2	45.9 ± 3.4	0.223
Total metabolizable^c^	75.8 ± 3.7	76.5 ± 3.9	0.862

Values are least‐squares means ± standard error.

a Control + rumen‐protected methionine.

b Estimated value.

c Absorbable rumen‐undegradable feed amino acid + Absorbable microbial amino acid.

### 3.5 Nitrogen balance

The N balance data are shown in Table 7. Input and output N did not differ between the two diet groups (*p *>* *0.10). The N partition ratio as a percentage of N intake did not differ (*p *>* *0.10).

**Table 7:** Nitrogen balance

Item	Diet		*p*
	Control	Treatment^a^	
Input and output of nitrogen, g/d			
Intake	557.8 ± 15.0	556.3 ± 15.3	0.907
Milk	202.6 ± 6.1	199.0 ± 6.5	0.688
Feces	216.6 ± 6.0	206.7 ± 6.4	0.272
Urine	159.0 ± 9.8	155.7 ± 10.2	0.724
Retention	−16.1 ± 14.5	−0.6 ± 15.6	0.421
Product^b^	187.4 ± 15.8	193.7 ± 16.8	0.787
Waste^c^	373.6 ± 11.2	363.2 ± 11.9	0.434
Nitrogen partition ratio, % of nitrogen intake			
Milk	36.3 ± 1.5	35.7 ± 1.5	0.688
Feces	38.8 ± 1.3	37.3 ± 1.4	0.337
Urine	28.6 ± 1.6	27.8 ± 1.7	0.697
Retention	−3.8 ± 2.4	−1.4 ± 2.6	0.479
Product^b^	32.8 ± 1.7	34.4 ± 1.9	0.556
Waste^c^	67.2 ± 1.7	65.6 ± 1.9	0.556

Values are least‐squares means ± standard error.

a Control + rumen‐protected methionine.

b Milk + retention.

c Feces + urine.

### 3.6 Health

The incidence of health disorders is shown in Table 8. The incidence of individual health disorders did not differ between dietary groups (*p *>* *0.05). The percentage of animals with mastitis tended to be higher in the Control group (*p *<* *0.10).

**Table 8:** Incidence of health disorders

Item	Diet		*p*
	Control	Treatment^a^	
Mammary, %			
Mastitis	30.0	5.3	0.092
Locomotive, %			
Leg problems	5.0	5.3	1.000
Hock problems	5.0	5.3	1.000
Digestive, %			
Milk fever	0.0	5.3	0.487
Ketosis	0.0	5.3	0.487
Displaced abomasum	0.0	5.3	0.487
Reduced appetite	25.0	15.8	0.695
Diarrhea	5.0	5.3	1.000
Reproductive, %			
Retained placenta	0.0	10.5	0.231

a Control + rumen‐protected methionine.

## 4 DISCUSSION

Our current results for DMI and milk production (Table 2) were not consistent with previous findings: in other studies, DMI (Schingoethe et al., 1988; Sun et al., 2016), milk yield (Batistel et al., 2017), 4% fat‐corrected milk (Sun et al., 2016), or yield or percentage of milk protein (Patton, 2010) changed when RPMet was supplemented. According to AminoCow, using the data from the production trial (DMI, milk yield, milk content of protein and fat), only the adequacies of Lys and Met were less than 110%. The adequacies in the Control and Treatment groups were, respectively, for Lys, 109% and 108%, and for Met, 96% and 104%. Only the Met requirement in the Control group was lacking (<100%) as designed, and supplementing with RPMet meant that the requirements for all essential amino acids were met. However, from our results we infer that feeding our 14.5% CP diet with RPMet does not affect milk production.

The results of the plasma analysis (Table 3) were within the normal reference ranges (Merck, 2012; Smith, 2009). The data on plasma glucose and nonesterified fatty acid concentrations agreed with the results reported by Socha et al. (2005), who found that these parameters were not affected when cows in early lactation were duodenally infused at 10.5 g/day with Met and 10.0 g/day with Lys. However, Sun et al. (2016) reported that RPMet supplementation at 15 g/day decreased the same parameters. Sun et al. (2016) also observed that total cholesterol was decreased by the addition of RPMet. In addition, Batistel et al. (2017) reported that plasma γ‐glutamyl transpeptidase levels decreased when cows were supplemented with 15 g/day of RPMet. From these previous reports and our results, we infer that some plasma items might change when diets contain RPMet at 15 g/day or more, but supplementation at the rate we used did not affect plasma profiles.

With the exception of the result for caproate, the ruminal fluid profiles (Table 4) were in agreement with the results of Armentano, Bertics, and Ducharme (1997), who reported that all ruminal fluid parameters were unaffected upon supplementation with RPMet at 10.5 g/day. On the other hand, Chung et al. (2006) reported in an in vitro experiment that the contents of total VFAs and some individual VFAs were altered by the addition of rumen‐protected Met. With the exception of the values for N, the apparent digestibility percentages (Table 5) were close to previous findings: dry matter and organic matter, (Ha & Kennelly, 1984; Miyaji, Matsuyama, Hosoda, & Nonaka, 2012), ether extract, NDFom and ADFom (Miyaji & Matsuyama, 2016; Miyaji et al., 2012). However, the results for apparent N digestibility were lower than in other reports. N digestibility has been reported as 67.6%–68.9% on a 15.5% CP diet (Miyaji et al., 2012) and 66.1% on a 13% CP diet (Ha & Kennelly, 1984). Our results support the suggestion by Ha and Kennelly (1984) that apparent N digestibility is elevated when dietary protein increases, because a higher protein content increases microbial fermentation in the rumen. From the results for the ruminal fluid profiling and digestibility, we can say that supplying RPMet did not affect ruminal fermentation.

The results for allantoin (Table 6) agree with those of Krober, Kulling, Menzi, Sutter, and Kreuzer (2000), who reported that urinary allantoin content did not differ between cows fed additional RPMet and those fed a control diet. Analysis of estimated metabolizable amino acids (Table 6) revealed that the intake of Lys did not differ between the diets, whereas the Met intake and amount of estimated absorbable rumen‐undegradable feed Met were significantly higher in the Treatment group, as planned. The amount of estimated total metabolizable Met did not differ between the two diet groups, because Met intake and the amount of estimated absorbable rumen‐undegradable feed Met were significantly higher in the Treatment group, whereas the amount of estimated absorbable microbial Met was (albeit nonsignificantly) lower in the Treatment group. It is unlikely that ruminal outflow of microbes was dramatically inhibited by the degradation of RPMet in the rumen, because the rate of ruminal disappearance of the Met in RPMet was as low as 18.9% and ruminal fermentation was not affected by supplying RPMet. We are unable to explain the cause of the trend toward a decrease in absorbable microbial Met in the Treatment group. Although we used the NRC (2001) data on the digestibility of amino acids in the intestine, some digestibility was reported (e.g., 75.7%, Berthiaume, Dubreuil, Stevenson, McBride, & Lapierre, 2001). More detailed work is needed to clarify this point. Regardless, supplying RPMet did not increase estimated total metabolizable Met in the metabolism trial. Moreover, the results of our plasma analysis and almost all of the results of our ruminal fluid profiling can be explained by assuming that total metabolizable Met during the production trial did not differ between groups. This conclusion is also supported by our findings that milk yield, milk composition, and milk component yield did not change with supplementation of RPMet. Consequently, the results of both the metabolism trial and the production trial suggest that total metabolizable Met did not differ between the groups.

The input and ouput results and the N partition ratios (Table 7) indicated that supplying RPMet on a 14.5% CP diet did not affect the N balance. In this connection, urinary N outputs in our cows were less than 159 g/day, although the milk yield was approximately 40.5 kg/day. Our previous studies have also shown that a diet including 14.5% CP and 5.0% RUP results in milk yields of more than 40 kg/day and in a urinary N content of more than 155 g/day during early lactation (unpublished data). Our urinary N outputs were lower than that in a previous report: the output was 177 g/day and the milk yield was 41.0 kg/day when cows were fed a diet including 16.3% CP (Miyaji & Matsuyama, 2016). According to JFS NARO, 2006, the recommended CP level in accordance with the results of our production trial (multiparous cows, body weight 660 kg, DMI 24.5 kg/day, milk yield 40.5 kg/day, milk fat 3.7%) is 15.5%; the level in our cows was thus about 1 percentage point lower. Thus a diet including approximately 14.5% CP—lower than recommended—can result in milk yields of 40 kg/day during early lactation. In contrast, dietary N that was not needed for amino acids for production is excreted as urinary N. We can therefore also conclude from the results for milk urea N (Table 2), plasma urea N (Table 3), and urinary N (Table 7) that feeding RPMet on a 14.5% CP diet does not improve the N balance under these experimental dietary conditions.

Mastitis (Table 8) was the only infectious disorder with a (nonsignificantly) higher prevalence in Control animals than in the Treatment group. Osorio et al. (2014) and Sun et al. (2016) reported that dietary supplementation with RPMet improves immune function. However, Batistel et al. (2017) found that the frequency of mastitis was unchanged when RPMet at 22 g/day was added, and Sun et al. (2016) found that the milk somatic cell count was unchanged when RPMet was fed at 15 g/day. Moreover, it is well known that mastitis is prevented mainly by using appropriate milking procedures. Consequently, we infer that the incidence of health disorders —including mastitis —was not altered by RPMet, because the amount of total metabolizable Met was similar in the dietary groups.

In conclusion, we found here that dietary supplementation with RPMet did not dramatically affect milk production, plasma profile, rumen fermentation, nitrogen balance, or cow health when cows during early lactation were fed approximately 14.5% CP, because the amount of total metabolizable Met did not change. We also demonstrated that milk yields of more than 40 kg/day can be achieved despite a decrease in the dietary CP level by about 1 percentage point from the 15.5% recommended by JFS and in the absence of supplemental RPMet.
